# Chinese Norms for the Autism Spectrum Rating Scale

**DOI:** 10.1007/s12264-017-0105-6

**Published:** 2017-02-24

**Authors:** Hao Zhou, Lili Zhang, Xiaobing Zou, Xuerong Luo, Kun Xia, Lijie Wu, Yimin Wang, Xiu Xu, Xiaoling Ge, Yong-Hui Jiang, Eric Fombonne, Weili Yan, Yi Wang

**Affiliations:** 1 Division of Neurology, Children’s Hospital of Fudan University, Shanghai, 201102 China; 2 Child Development Center, The Third Affiliated Hospital, Sun Yat-Sen University, Guangzhou, 510000 China; 3 Department of Psychiatry, The Second Xiangya Hospital of Central South University, Changsha, 410008 China; 4 State Key Laboratory of Medical Genetics, Changsha, 400078 China; 5 School of Public Health, Harbin Medical University, Harbin, 150081 China; 6 Department of Child Healthcare, Children’s Hospital of Fudan University, Shanghai, 201102 China; 7 Children’s Hospital of Fudan University, Shanghai, 201102 China; 8 Division of Medical Genetics, Department of Pediatrics and Neurobiology, Duke University School of Medicine, Durham, NC 27710 USA; 9 Oregon Health and Science University, Portland, OR 97239 USA; 10 Department of Clinical Epidemiology, Children’s Hospital of Fudan University, Shanghai, 201102 China; 11 Pediatric Department, Guizhou Provincial People’s Hospital, Guiyang, 550002 China

**Keywords:** Autism spectrum disorders, Autism spectrum rating scale, Norm, Children

## Abstract

This study aimed to establish norms for the modified Chinese version of the Autism Spectrum Rating Scale (ASRS). Participants were recruited from Shanghai, Harbin, Guangzhou, and Changsha, China, and their parents and teachers were invited to complete the Chinese Parent version and the Teacher version of the ASRS. In both versions, boys had significantly higher sub-scale scores and total score (T-score) by 1–3 and 4–5 points respectively, than girls (both *P* < 0.001). Age had weak correlations with some sub-scores and the T-score (*r* ranged from −0.1859 to 0.0738), and some reached significance (*P* < 0.03). The correlations appeared stronger and were more common in females. The T-score based on Chinese norms ideally correlated with the score based on the United States norms in boys and girls for both versions. Norms for the Chinese version of the ASRS for children aged 6–12 years are proposed and may be helpful for screening individuals with autism spectrum disorders from the general population of children.

## Introduction

Autism spectrum disorders (ASDs) are a set of heterogeneous neurodevelopmental conditions characterized by early-onset developmental impairments in social communication and unusually restricted, repetitive behaviors and interests [1]. Epidemiological studies have identified various risk factors, but none has been shown to be necessary or sufficient for the development of autism [2]. Understanding of the gene-environment interplay in autism is still at an early stage and needs further research. Meta-analysis [3] has shown that individuals with autism have a mortality risk 2.8-times higher than that of unaffected people of the same age and gender. Higher childhood intelligence, communicative phrase speech before age 6, and fewer childhood social impairments predict a better outcome. Yet, even for individuals without intellectual disabilities, the outcome of social communication in adulthood is often unsatisfactory in terms of quality of life and achievement of occupational potential [4].

The global prevalence of autism has increased 20- to 30-fold since the earliest epidemiologic studies were conducted in the late 1960s and early 1970s, and now the reported worldwide population prevalence is ~1% [5], which is a major concern for those who care for affected children and their families. The Centers for Disease Control and Prevention (USA) set up the Autism and Developmental Disabilities Monitoring network to periodically monitor ASD prevalence [6]. In China, the official prevalence of ASD is not yet known. Estimates can be obtained from large-scale surveys, but care should be taken when selecting screening instruments based on various characteristics of the target sample and on the purpose of a study.

Several scales exist to screen for autistic traits in the general population, including the Social Communication Questionnaire (developed in 1999) [7], the Autism Spectrum Screening Questionnaire (developed in 1999) [8], the Autism Spectrum Quotient (developed in 2001) [9], the Childhood Autism Screening Test (developed in 2002) [10], and the Social Responsiveness Scale (developed in 2003) [11]. The Autism Spectrum Rating Scale (ASRS) is a newer scale developed in 2009 to identify youths who are most likely to need additional evaluation or services for ASD and related issues [12]. In this study, we constructed ASRS norms based on several advantages of the scale as a screening tool for ASD. First, the ASRS is not just a screening tool; it is also helpful in guiding diagnostic decisions and can be used for treatment planning, ongoing monitoring of the response to intervention, and program evaluation. In addition, the ASRS was designed for both young children aged 2–5 years and youths aged 6–18 years, from a diverse group of individuals.

Finally, comparisons with other instruments are easy due to the availability of standard scores. As the prevalence of ASD and the risk of over- and under-diagnosis are increasing in China, a valid, reliable, and carefully-crafted tool for screening and treatment assessment is needed. The norms of a Chinese version of the ASRS are expected to meet this need [12]. Previous studies have also shown that the cultural setting can impinge on the performance of scales [13, 14]. Therefore, the aim of this study was to propose Chinese norms, before its application for national screening in the general Chinese population.

## Methods

### Study Population

This study was conducted from January to July, 2014. Community-based participants (aged 6–12 years) were selected as the general sample to ensure its representativeness by using convenient cluster sampling. Four community-based samples were selected from Shanghai, Harbin, Guangzhou, and Changsha. From each site, one administrative street containing >400 children aged 6–12 years was chosen; all children with residency were recruited and comprised the reference sample.

The protocol was approved by the Research Ethics Committee of the Children’s Hospital of Fudan University. Both the parental and teachers’ consent was in written form.

### Measurements

#### Description of the Instrument

The ASRS contains screening, DSM-IV-TR, and treatment scales, with a total of 71 items. The screening scale comprises 60 items of the total 71 including Social/Communication (SC), Unusual Behaviors (UB), and Self-Regulation (SR). The DSM-IV-TR scale contains 34 items of the total 71 and a higher score indicates a higher chance of a diagnosis of autism by a psychiatrist. The treatment scale has 69 items of the total 71 and includes 8 scales.

Each scale yields a raw score by summing the relevant items. This raw score is subsequently transformed into a standardized score with a mean of 50 and a standard deviation (SD) of 10. The T-score incorporates the information from the 3 screening scales; the 3 standardized scale scores are first summed, and then transformed into a single score with a mean of 50 and a SD of 10. In that way, each of the screening scales contributes an equal weight to the overall summary T-score. Further details of the scoring procedures and interpretation of scores can be found in the ASRS manual [12].

#### Development of the Chinese Version of the ASRS

A pilot study was first conducted to establish the reliability and validity of the Chinese ASRS, and they were found to be excellent. As in the original research in the USA that developed the ASRS, we conducted an exploratory factor analysis to confirm the factor structure of the ASRS in a Chinese sample; this can be found in a companion paper entitled “Modifying the Autism Spectrum Rating Scale (6–18 years) to a Chinese Context: An Exploratory Analysis” in this issue [15]. Based on the same selection criteria of factor loading, >0.30, our analysis retained 59 items (as compared to 60 in the US study) loading on a comparable 3-factor structure. The content of the 3 factors was similar to that of the original US study, and therefore the factor names were retained. The only difference was that the numbers of items for each factor were different in the China validation sample, with SC, UB, and SR now having 21, 14 and 24 items. The DSM-IV-TR scale was based on expert judgment as to which items in the ASRS closely map each of the diagnostic criteria for PDD. Therefore, the DSM-IV-TR scale was used as recommended in the original US manual.

### Procedure

With the approval of Multi-Health Systems, we prepared a Chinese version of the ASRS by the usual translation-back-translation approach, and the pilot study allowed us to confirm the linguistic appropriateness [16]. Researchers were trained before the scales were distributed. Most parents were asked to complete the questionnaire at home, and they were subsequently collected by researchers. Other questionnaires completed by parents were collected by the teachers in a sealed envelope. Teacher ratings were collected directly from the school. Parents and teachers completed the scales at the same time.

Basic personal information about the child’s date of birth, gender, and school was requested. The child’s age was calculated as the difference between the date of questionnaire completion or return and the birth date. Rating scores were excluded if the child was older or younger than the target age-range. All scores were entered online using a database created from the original scoring method. Quality control of the data was performed before further analysis.

### Quality Control

A detailed schedule for data collection was developed and implemented in the four sites. All research staff was trained in the administration and scoring of the questionnaires. To facilitate data entry and checks, we established an online multi-center database that was accessible to the teams at each center to promptly upload and check data. All rating scores were scrutinized for errors or missing information. Before data analysis, a few parental ASRS questionnaires were excluded for reasons including errors on the birth date and an older or younger than the target age range. Analyses were subsequently performed with or without the excluded questionnaires.

### Statistical Analysis

Data analyses were performed using Stata 11 software (version 11.0, College Station, TX). Conventional descriptive analyses were used to present the site and gender distribution of the study sample, and the differences in raw score distributions of the three factors SC, SR, and UB. Student’s *t* test was used to test for gender differences. Analyses of variance (ANOVA) were used to examine differences among sites. Multiple linear regression analyses were used to assess the effects of gender, age and site on ASRS scores. Participants aged 6–12 years were treated as one age group. All ASRS subscale scores and T-scores were normalized to a normal distribution with a mean of 50 and standard deviation of 10. The agreement of the T-score normal distribution for the Chinese population with that for US norms was tested by Pearson correlation analysis. All *P* values were two-sided and *P* values <0.05 were deemed statistically significant.

## Results

In this study, 2053 children were eligible for inclusion in the general sample. After exclusion of questionnaires due to various errors, 1625 parental questionnaires were available for the normative sample (830 boys and 795 girls; mean age, 8.85 ± 1.78 years). In addition, after exclusion of questionnaires with various errors, 1514 teacher questionnaires were finally available (772 boys and 742 girls; mean age, 8.96 ± 1.75 years). All teachers or caregivers had known the students for at least 1 month.

Demographic characteristics of the sample are shown in Tables 1 and 2. The participants’ age and gender did not differ significantly between the parent and teacher groups.

**Table 1:** Age and gender distribution of the reference sample.

Age	Parent rating			Teacher rating		
	Male *n* (%)	Female *n* (%)	Total	Male *n* (%)	Female *n* (%)	Total
6	84 (53.16)	74 (46.84)	158	56 (52.34)	51 (47.66)	107
7	164 (52.23)	150 (47.77)	314	160 (55.94)	126 (44.06)	286
8	128 (51.61)	120 (48.39)	248	117 (49.16)	121 (50.84)	238
9	155 (52.36)	141 (47.64)	296	146 (50.34)	144 (49.66)	290
10	109 (45.80)	129 (54.20)	238	111 (48.47)	118 (51.53)	229
11	125 (50.20)	124 (49.80)	249	111 (47.03)	125 (52.97)	236
12	65 (53.28)	57 (46.72)	122	71 (55.47)	57 (44.53)	128
Total	830 (51.08)	795 (48.92)	1625	772 (50.99)	742 (49.01)	1514
*χ* ^*2*^	4.0905			6.3404		
*P*	0.664			0.386

**Table 2:** Gender distribution of the reference sample by study site.

City	Parent rating		Teacher rating	
	Male *n* (%)	Female *n* (%)	Male *n* (%)	Female *n* (%)
Shanghai	216 (49.32)	222 (50.68)	203 (46.77)	231 (53.23)
Guangzhou	228 (52.29)	208 (47.71)	227 (52.06)	209 (47.94)
Changsha	182 (51.12)	174 (48.88)	187 (52.53)	169 (47.47)
Harbin	204 (51.65)	191 (48.35)	155 (53.82)	133 (46.18)
Total	830 (51.08)	795 (48.92)	772 (50.99)	742 (49.01)
*χ* ^*2*^	0.5963		4.5476	
*P*	0.897		0.208	

In the parent and teacher versions of the ASRS, boys had significantly higher raw scores in SC, UB, SR, T-score, and DSM-TR by 1–3 for parents rating, and 4–5 points for teachers than girls (*P* < 0.001). Age showed a weak correlation with some sub-scores and T-score (*r* ranged from −0.1859 to 0.0738), and some were significant (*P* < 0.03; Table 3). The correlations were stronger and more common in females. ANOVA revealed slight site differences in the raw scores of subscales (Table 4).

**Table 3:** Pearson correlation analyses of ASRS scale scores with age by gender.

Sub-scales	Male		Female	
	r	*P*	r	*P*
Parent rating				
SC_R	0.0738	0.0342	0.0305	0.3926
UB_R	−0.0250	0.4722	−0.1075	0.0025
SR_R	−0.0967	0.0054	−0.1859	<0.0001
T-score	−0.0113	0.7468	−0.0987	0.0057
DSM_R	0.0114	0.7429	−0.0543	0.1269
Teacher rating				
SC_R	−0.0638	0.0779	−0.1162	0.0016
UB_R	−0.0093	0.7960	−0.0524	0.1536
SR_R	−0.0882	0.0149	−0.1341	0.0002
T-score	−0.0607	0.0954	−0.1303	0.0004
DSM_R	−0.0724	0.0449	−0.1545	<0.0001

*DSM* diagnostic and statistical manual of mental disorders, *r* correlation coefficient, *R* raw score, *SR* self-regulation, *SC* social/communication, *T-score* standardized total score, *UB* unusual behaviours.

**Table 4:** Site and gender differences in raw sub-scale scores and T-score.

Sub-scales	City				Gender	
	Shanghai *n* = 438	Guangzhou *n* = 436	Changsha *n* = 356	Harbin *n* = 395	Male *n* = 830	Female *n* = 791
Parent rating						
SC_R	27.53 ± 12.71	36.98 ± 13.28	32.18 ± 13.07	26.04 ± 14.07	31.80 ± 14.09	29.61 ± 13.76
UB_R	29.32 ± 10.39	31.78 ± 11.02	32.70 ± 9.44	26.68 ± 10.49	30.74 ± 10.58	29.38 ± 10.65
SR_R	20.43 ± 8.84	24.21 ± 9.19	22.69 ± 7.93	18.34 ± 8.44	22.92 ± 9.02	19.86 ± 8.56
T-score	48.26 ± 9.98	53.75 ± 9.61	52.16 ± 8.49	45.88 ± 9.73	51.29 ± 10.00	48.65 ± 9.83
DSM_R	39.78 ± 12.98	46.26 ± 12.30	44.21 ± 11.68	37.28 ± 13.10	43.29 ± 12.99	40.40 ± 12.94
Teacher rating						
SC_R	31.61 ± 17.15	40.01 ± 16.05	33.55 ± 17.56	27.04 ± 16.76	36.37 ± 17.51	30.82 ± 16.98
UB_R	27.00 ± 11.73	27.22 ± 12.19	31.18 ± 11.38	27.26 ± 12.04	29.54 ± 12.12	26.59 ± 11.60
SR_R	20.42 ± 11.21	23.23 ± 10.74	21.81 ± 10.22	19.13 ± 10.71	24.02 ± 10.69	18.54 ± 10.31
T-score	48.91 ± 10.23	52.05 ± 9.90	51.15 ± 9.44	47.03 ± 9.58	52.06 ± 9.93	47.89 ± 9.63
DSM_R	40.15 ± 15.84	45.44 ± 15.88	43.39 ± 15.27	37.20 ± 15.74	44.80 ± 16.13	38.88 ± 15.25

*R* raw score, *SR* self-regulation, *SC* social/communication, *T-score* standardized total score, *UB* unusual behaviours.

**Fig. 1:**
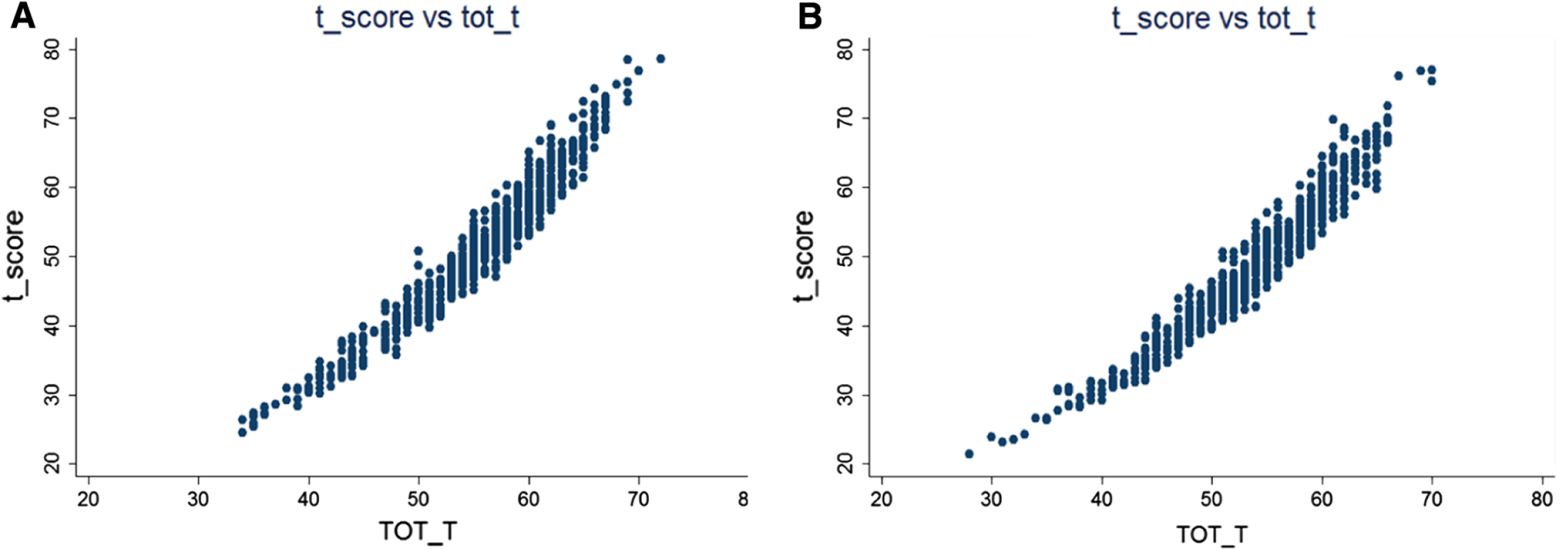
Correlations between the T-score based on the Chinese norm and that based on the US norm (parent ratings) for boys (**A**), and girls (**B**) for the parent version. t_score, T-score calculated by Chinese norm; tot_t, T-score calculated based on the US norm. *r* = 0.9674, *P* < 0.001 for boys and *r* = 0.9664, *P* < 0.001 for girls.

**Fig. 2:**
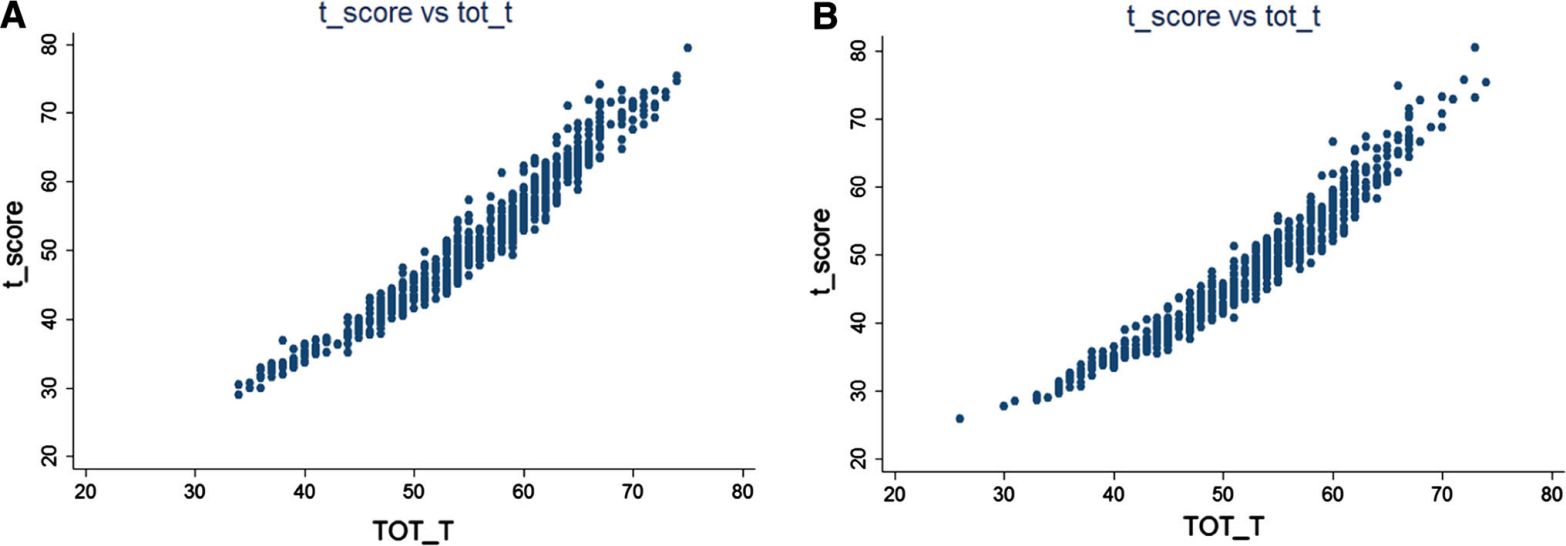
Correlations between the T-score based on the Chinese norm and that based on US norm (teacher ratings) for boys (**A**) and girls (**B**) for the teacher version. t_score, T-score calculated by Chinese norm; tot_t, T-score calculated based on the US norm. *r* = 0.9715, *P* < 0.001 for boys and *r* = 0.9683, *P* < 0.001 for girls.

The T-score of the reference sample showed a significant correlation with T-scores that were computed based on American ASRS norms for the parent version (*r* = 0.9674 for boys and 0.9664 for girls, *P* < 0.001; Fig. 1A, B). For the teacher version, the correlation coefficient values were 0.9715 and 0.9683, respectively (Fig. 2A, B).

## Discussion

In this study, we obtained norms for the ASRS sub-scales and T-score from a culturally and linguistically representative community-based sample of Chinese 6–12 year-old children, which could be used to determine which young people are most likely to require additional evaluation or services for ASD and related issues.

We found that gender had a significant effect on SC, UB, SR, T-score, and DSM-IV-TR as both parents and teachers rated males higher than females. This supports previous evidence that boys and girls have social and communication developmental trajectories with different profiles, boys typically displaying higher levels of difficulty in social and communicational skills. These gender differences are consistent with research findings that ASDs occur far more frequently in males than in females with a prevalence ratio of 4.5:1 [5, 6, 17, 18]. Many researchers have focused on mechanisms that explain the contribution of gender differences to the risk of ASDs [19, 20] such as the Extreme Male Brain theory [21–24]. However, further research is needed to fully understand the origins of this robust difference.

The results of the current study show minor age effects in the ASRS scores for both parental and teacher ratings, which is consistent with the findings in the ASRS norm study [12], indicating that developmental trends in the scores are very small. Despite the fact that initial signs and symptoms typically emerge in the early developmental period, consistently before age 3, some social deficits and behavioral patterns might not be recognized as symptoms of ASD until a child is unable to meet social, educational, occupational, or other important life-stage demands [25]. This finding supports the proposal that the norm is to cover Chinese children aged 6–12 years.

Although the representativeness of the reference samples for developing norms was ensured by including 4 cities in China, Shanghai, Guangzhou, Changsha, and Harbin, cultural and economic differences may exist. Uniform protocols were applied for data collection. The results showed balanced age and gender distributions of the 4 sub-samples; however, mean raw subscale scores and standard deviations showed slight differences among the 4 cities. This may reflect sample differences across the 4 sites. As we did not have individual data on respondents with regard to profession, education, or other variables that may influence scores, we were unable to further investigate the source of these differences. However, the 4 sites were selected in regions that differ slightly with respect to cultural background and level of economic development. It is likely that these differences reflect true variability in the population that was appropriately reflected in our normative sample. After statistical normalization, combination of the 4 sub-samples helped to enhance the representativeness of the reference study sample.

Based on exploratory factor analysis, we made slight changes to items and structure of the scales (refer to the companion paper entitled “Modifying the Autism Spectrum Rating Scale (6–18 years) to a Chinese Context: An Exploratory Analysis” [15]). The present data provide encouraging evidence in support of use of the ASRS, given an excellent positive correlation with the US norm data. This shows that the slightly-modified ASRS is suitable for screening ASD in the Chinese cultural environment.

One limitation of this study is that we only selected urban populations as the reference sample in this study, and it was relatively limited. Therefore, it is necessary to include rural populations in further studies.

In conclusion, we have established the Chinese norm referenced criteria for ASRS, adopting the theoretical approach used for other languages and settings. The excellent correlation between our normative data and those in the USA demonstrated the high quality of this scale. The normative data will be useful in the screening and clinical evaluation of school-aged children in China.
